# REV, A BRET-Based Sensor of ERK Activity

**DOI:** 10.3389/fendo.2013.00095

**Published:** 2013-07-30

**Authors:** Chanjuan Xu, Marion Peter, Nathalie Bouquier, Vincent Ollendorff, Ignacio Villamil, Jianfeng Liu, Laurent Fagni, Julie Perroy

**Affiliations:** ^1^CNRS, UMR-5203, Institut de Génomique Fonctionnelle, Montpellier, France; ^2^INSERM, U661, Montpellier, France; ^3^UMR-5203, Universités de Montpellier 1 & 2, Montpellier, France; ^4^Sino-France Laboratory for Drug Screening, Key Laboratory of Molecular Biophysics of Ministry of Education, College of Life Science and Technology, Huazhong University of Science and Technology, Wuhan, China; ^5^CNRS, UMR 5535, Institut de Génétique Moléculaire de Montpellier (IGMM), Montpellier, France; ^6^UMR866 Dynamique Musculaire et Métabolisme, INRA, Université Montpellier 1, Université Montpellier 2, Montpellier, France

**Keywords:** biosensor, bioluminescence resonance energy transfer, BRET imaging, fluorescence lifetime imaging microscopy, extracellular signal-regulated kinases, spatio-temporal signaling, Rluc8-ERKsubstrate-Venus

## Abstract

Networks of signaling molecules are activated in response to environmental changes. How are these signaling networks dynamically integrated in space and time to process particular information? To tackle this issue, biosensors of single signaling pathways have been engineered. Bioluminescence resonance energy transfer (BRET)-based biosensors have proven to be particularly efficient in that matter due to the high sensitivity of this technology to monitor protein–protein interactions or conformational changes in living cells. Extracellular signal-regulated kinases (ERK) are ubiquitously expressed and involved in many diverse cellular functions that might be encoded by the strength and spatio-temporal pattern of ERK activation. We developed a BRET-based sensor of ERK activity, called Rluc8-ERKsubstrate-Venus (REV). As expected, BRET changes of REV were correlated with ERK phosphorylation, which is required for its kinase activity. In neurons, the nature of the stimuli determines the strength, the location, or the moment of ERK activation, thus highlighting how acute modulation of ERK may encode the nature of initial stimulus to specify the consequences of this activation. This study provides evidence for suitability of REV as a new biosensor to address biological questions.

## Introduction

The specificity of cellular responses to receptor stimulation is encoded by the spatial and temporal dynamics of downstream signaling networks. Cells indeed respond to multiple external stimuli thanks to a surprisingly limited number of signaling pathways activated by plasma membrane receptors. To encode and make distinct various external signals these commune pathways have to be precisely regulated in space and time. Thus distinct spatio-temporal activation profiles of a shared repertoire of signaling proteins result in different gene activation patterns and diverse physiological responses (1–3).

An emerging picture of interrelated networks has therefore superseded the former preconceived scheme of discrete linear pathways to convey extracellular signals to specific targets. In fact, various receptor pathways share a common protein catalog that mediates signal transduction. For any individual receptor pathway, there is no single protein or gene responsible for signaling specificity. Rather, specificity is determined by the temporal and spatial dynamics activation of downstream signaling components. To address and answer the questions surrounding the specificity of signal to response events, signaling reporters for individual downstream signaling components activation in intact cellular environment will be required.

In recent years, efforts have been made to study the dynamic cellular processes by the engineering of biosensors specific to various signaling pathways. Resonance Energy Transfer technologies (Fluorescent, FRET or Bioluminescence resonance energy transfer, BRET) have proven to be efficient in this area by enabling the monitoring of protein–protein interactions or protein-conformational changes in living cells. FRET and BRET technologies are both based on the non-radiative transfer of energy between the donor and acceptor molecules via the Förster mechanism and primarily depend on: (1) an overlap between the emission and excitation spectra of the donor and acceptor molecules, respectively; and (2) the close proximity of the donor and acceptor entities (<100 Å) (4, 5). In the case of FRET, both the donor and acceptor are fluorescent molecules, whereas in BRET, the energy donor is a bioluminescent molecule. FRET necessarily requires fluorescence excitation, resulting in problems of photobleaching, autofluorescence, simultaneous excitation of both donor and acceptor fluorophores, phototoxicity, and undesirable stimulation of photobiological processes. Most of these FRET drawbacks tend to be corrected with the emergence of technologies like TR-FRET or Fluorescence Lifetime Imaging Microscopy (FLIM) (6, 7). Nevertheless, the use of BRET allows these practical problems to be bypassed, as it is initiated by an enzymatic reaction (instead of fluorescence excitation). Accordingly, bioluminescence-initiated resonance energy transfer results in greater sensitivity in living subjects because of a higher signal to background ratio, and therefore makes BRET a technology of choice for measurements from cell lysates or intact cells (8).

The Mitogen-Activated Protein Kinases (MAPK) family is a class of serine/threonine kinases [including the Extracellular signal-regulated kinases (ERK), p38, and JNK sub-families] that are ubiquitously expressed, activated by various stimuli and therefore involved in numerous cellular functions. The ERK signaling cascade is a central MAPK pathway that plays a role in the regulation of various cellular processes such as proliferation and differentiation, development, neuronal plasticity and learning, survival, and apoptosis (9, 10). The ability of this cascade to regulate so many distinct and even opposing cellular processes raises the question of signaling specificity determination by this cascade. Duration and strength of the signals, interaction with specific scaffolds, changes in sub-cellular localization, crosstalk with other signaling pathways, and presence of multiple components with distinct functions in each tier of the cascade seems to determine the ultimate function of ERK activation (11–14). The reliability of signaling and the spatio-temporal activation of ERK are therefore key context-dependent determinants that need to be deciphered in order to resolve the nature of precise biological responses.

To better understand the final outcome of intricate factors controlling the kinetics of ERK activity, signaling reporters in living cells have been engineered (15–18). In the present work we used the most efficient biosensor for ERK activity so far tested (18). We reproduced published data, however the sensitivity of this FRET biosensor was not sufficient to detect subtle variations of ERK activity in neurons. We therefore improved this biosensor by switching the FRET tags to BRET compatible entities. We then defined the proper experimental conditions to record accurate BRET signals with this ERK-biosensor, described the potential and limitation of this upgraded reporter, and discussed possible further improvements. This work also addresses general technical concerns about the use of biosensors.

## Materials and Methods

### Reagents

Glycine (200 μM), strychnine (1 μM), GABA (100 μM), U0126 (10 μM), PMA (1 μM), and KCl (50 mM) were all purchased from Sigma-Aldrich, St Quentin Fallavier, France. NMDA (50 μM) was purchased from Tocris (Fisher-Bioblock, Illkirch, France) and epidermal growth factor (EGF) (50 ng/ml) from Calbiochem (MerckMillipore, Darmstadt, Allemagne). We used the following primary antibodies: p44-42 MAP Kinase Antibody (Cell Signaling Technology, #9102), phospho-p44p42 MAP Kinase (Thr202/Tyr204) antibody (Cell Signaling Technology, #9101), p-Thr-48-Cdc25C antibody (Cell Signaling Technology, #9527), and rabbit GFP antibody (Invitrogen, A11122).

### REV construction

Plasmids coding for nuclear and cytoplasmic Extracellular signal-regulated Kinase Activity Reporter (EKAR), pRK5-Cerulean-EKAR_Nucl_-Venus, and pRK5-Cerulean-EKAR_cyto_-Venus (18) (Addgene, Cambridge, MA, USA) were digested by *Cla*I and *Bam*HI restriction enzymes to remove the Cerulean-coding sequence and replace it by the Rluc8-coding sequence amplified by PCR between *Cla*I and *Bam*HI restriction sites. We thus obtained two plasmids: pRK5-Rluc8-EKAR_Nucl_-Venus and pRK5-Rluc8-EKAR_cyto_-Venus. Inactive mutants were made by mutation in EKAR Cdc25C peptide “PDVPRTPVGK” (Thr-to-Ala substitution). The control pRK5-Rluc8-EKAR (cyto) plasmid was designed from the pRK5-Rluc8-EKARcyto-Venus construct: the original Rluc8-ERKsubstrate-Venus (REV) sequence was removed by a *Cla*I/BrsGI enzymatic digestion and replaced with a Rluc8-EKAR sequence obtained by PCR on pRK5-Rluc8-EKARcyto-Venus with insertion of *Cla*I/BrsGI appropriate restriction sites.

### Cell cultures and transfection

HEK293T cell culture and calcium phosphate transfection were performed as previously described (19). To determine the optimal level of REV expression we performed many transfections with different amounts of pRK5-Rluc8-EKAR_Nucl_-Venus and pRK5-Rluc8-EKAR_cyto_-Venus plasmids, ranging from 0.1 ng to 4 μg of each plasmid per 100 mm diameter cell dish (3,000,000 cells). The total amount of DNA per plate dish was complemented with the non-coding plasmid pcDNA3 to reach 5 μg of DNA in each transfection. We chose the transfection condition containing 20 ng of REV-coding plasmids for the other experiments. Hippocampal neuronal primary cultures were prepared from 17.5 days embryonic mice (E17.5) and grown in neurobasal medium (Gibco, Invitrogen, Cergy Pontoise, France) supplemented with 2% B-27 (Gibco), glutamax (4 mM, Gibco), glutamic acid (25 μM, Gibco), antibiotics (Penicillin 100 U/ml and Streptomycin 100 μg/ml), and 10% Fetal Bovine Serum (FBS), in 35 mm diameter glass bottom culture dishes (MatTek Corporation, Ashland, MA, USA). After 3 days in culture (DIV3), the culture medium was supplemented with Cytosine β-d-arabinofuranoside hydrochloride 5 μM (Sigma-Aldrich, St Quentin Fallavier, France) for 12 h. Then, 75% of the medium was replaced by neurobasal medium supplemented with B-27, glutamax, and antibiotics. Neurons were then transfected with 100 ng of pRK5-Rluc8-EKAR_Nucl_-Venus and pRK5-Rluc8-EKAR_cyto_-Venus plasmids and 1.8 μg of the non-coding plasmid pcDNA3 using Lipofectamine 2000 (Invitrogen, Cergy Pontoise, France) according to the manufacturer’s standard protocol at DIV10 and studied between DIV11 and DIV12.

### Immunofluorescence staining and imaging

Immunofluorescence staining was performed on HEK cells, transfected or not with REV, were fixed with 4% PFA for 10 min at room temperature, permeabilized with Triton X-100 0.15% for 10 min, washed and incubated with blocking buffer (FBS 10%, BSA 1% in PBS) for 2 h at RT. Polyclonal antibodies raised against p44-42 MAP kinase were incubated overnight at 4°C in PBS containing 1% BSA. After three rinses with PBS, the anti-mouse Cy3-conjugated antibodies (1:500, Jackson) were added for 30 min at RT. Three rinses with PBS were carried out before mounting cells directly in the wells under cover slips.

Images were obtained with LSCM (OLYMPUS, FV-1000, 60× objective), equipped with appropriate epifluorescence and filters (Green: 475_40 and 530_50 nm for excitation and emission respectively, Red: 545_25 and 605_70 nm for excitation and emission respectively). Images were digitized and saved in TIFF format using the Andor software and further analyzed using the ImageJ software (NIH).

### Western blots

Cells were lysed in 0.1% Triton X-100, 150 mM NaCl, 2 mM EGTA, anti-protease mixture (Roche Applied Science), phosphatase inhibitors (Na_3_VO_4_, NaP_2_O_3_, NaF), and 20 mM Tris-HCl, pH 7.4 (lysis buffer), and the mixture was centrifuged. The supernatant was incubated in a Laemmli buffer at 90°C. Proteins were transferred to nitrocellulose (NC) membranes (Millipore, Bedford, MA, USA) and blocked in blocking buffer (5% non-fat dry milk in TBS and 0.1% Tween 20) for 1 h. The blots were then incubated with primary antibodies at the relevant dilution (Cell Signaling Technology, Beverly, MA, USA) for 1 h at room temperature, and with horseradish peroxidase-linked secondary antibodies (1:20,000; Pierce, USA) for 2 h. Immunoblots were revealed using the enhanced chemiluminescence reagents (Thermo, USA) and visualized using the X-ray film. The density of immunoreactive bands was measured using NIH image software, and all bands were normalized to percentages of control values.

### Fluorescence lifetime imaging microscopy

Time-domain FLIM was performed with a multiphoton microscopy system, based on a Zeiss Axiovert 200M LSM 510 Meta NLO equipped with a Ti:Sapphire Chameleon-XR pulsed laser (Coherent). Time-resolved detection was afforded by the addition at a non-descanned output of a fast photomultiplier and SPC-830 time-correlated single-photon counting (TCSPC) electronics (7). For EGFP excitation, laser power at 900 nm was adjusted to give average photon counting rates of the order 10^4^–10^5^ photons s^−1^ (0.0001–0.001 photons/excitation event) and with peak rates approaching 10^6^ photons s^−1^, below the maximum counting rate afforded by the TCSPC electronics to avoid pulse pile-up. Acquisition times of 120 s were used. Analysis of the fluorescent transients was performed with the SPCImage software package (7). Images were taken with a Zeiss 63×/1.0 W Plan-Apochromat objective.

### BRET measurements

Bioluminescence resonance energy transfer measurements in cell populations were performed as previously described (19). Single cell BRET imaging in cultured hippocampal neurons to study the sub-cellular localization of REV-conformational changes were performed according to previous protocols (20, 21). Briefly, images were obtained using a Plan-Apochromat 63×/1.40 Oil M27 objective, at room temperature. Hippocampal neurons were transfected at DIV10 and recorded at DIV11 or DIV12 in the following external medium (in millimolar): 140 NaCl, 0.5 CaCl_2_, 3 KCl, 10 HEPES, 10 d-Glucose, 0.0003 tetrodotoxin, pH 7.4 and osmolarity of 330 mOsm. Transfected cells were first identified using a monochromatic light and appropriate filter to excite Venus (exciter HQ480/40 #44001 – emitter HQ600/50 #42017, Chroma). The light source was then switched off until the end of the experiment. Coelenterazine H (CoelH, 20 μM) was applied for 5 min before acquisition with Metamorph software (Molecular Devices). BRET images were collected every 30 s by sequential acquisitions from the 535 and 480 nm channels of 7 s each, using the evolve camera from Photometrics. Drugs were added 2 min (or four images) after the first acquisition. Sequential acquisitions were performed at 5 MHz (Gain 3950, binning 1) with emission filters D480/60 nm (#61274, Chroma) and HQ535/50 nm (#63944, Chroma) to select em480 and em535 wavelengths respectively. We applied an exclusive threshold on the em480 image, from 0 to 4,000 counts, in order to exclude these non-reliable weak values (see Figure 3A). The pixel-by-pixel 535/480 nm ratios were calculated by dividing the absolute blue or yellow intensities per pixel of images obtained at 535 nm over 480 nm. These numerical ratios (comprised between 0 and 1.5) were translated and visualized with a continuous 256 pseudo-color look-up table (LUT) as displayed in the figures. We determined the average intensity (“mean”) and distribution (“Standard Deviation”) of the 535/480 nm fluorescence ratios, in a square region of pixels drawn on the sub-cellular compartment of interest using Image J software (NIH). The “mean” measured the global BRET intensity in that area, while the “Standard deviation” of BRET from pixel to pixel gave information about the distribution of the BRET signals in that area. These are two complementary pieces of information. We then averaged the mean ± SEM and Standard Deviation ± SEM obtained from three to six cells and seven square regions per cell in the same sub-cellular compartment and in identical stimulating conditions. Please note that a high standard deviation indicates a spatial clusterization of the BRET signals and not a variation of mean between similar areas, this latter being detected by the “SEM” of the mean.

### Statistical analyses

Analyses were performed using Prism software. Statistical analyses were performed with the non-parametric Kruskal and Wallis test for more than two independent samples or with Friedman test for paired samples with a “*p*” risk threshold of 5%.

## Results

### Construction of “REV,” a BRET-based sensor of ERK activity

Extracellular signal-regulated Kinase Activity Reporter is a FRET-based sensor of ERK activity optimized for signal-to-noise ratio and FLIM (18). Briefly, the ERK activity sensor includes a substrate phosphorylation peptide from Cdc25C containing the consensus MAPK target sequence (PRTP) (22), and the proline-directed WW phospho-binding domain (23) boxed between FRET compatible entities (EGFP and mRFP1). Phosphorylation of the substrate sequence by ERK activation induces the binding of the phospho-binding domain and subsequent conformational rearrangement, thus triggering a change in FRET between the donor and acceptor entities. Because specificity in MAPK signaling depends on docking domains (24), EKAR contains an ERK specific docking site (FQFP) next to the phosphorylation sequence (25). Finally, a central flexible linker consisting of 72 glycine residues spaces out the phospho-binding domain from the substrate peptide to allow conformational changes (18).

**Figure 1:**
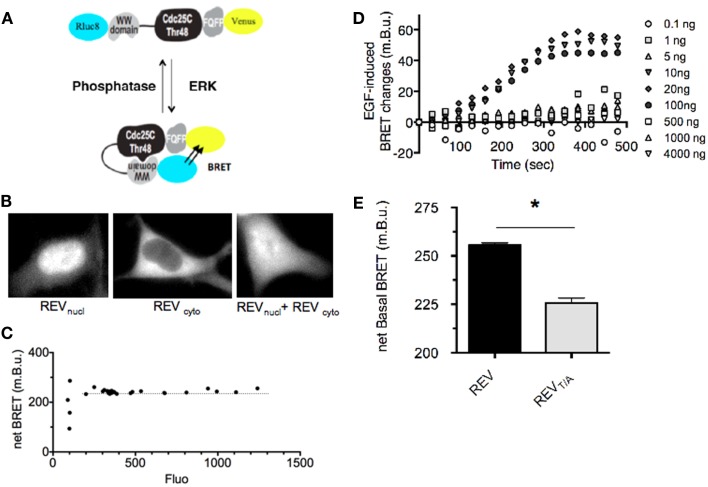
**Design and characterization of REV, a BRET-based sensor of ERK activity**. **(A)** Schematic representation of ERK sensor-conformational changes induced upon ERK activation, adapted from Harvey et al. (18). The conformational change induced by REV-phosphorylation increases the proximity between Rluc8 and Venus, promoting BRET-increase. **(B)** Fluorescence of REV_nucl_ and REV_cyto_ transfected alone or together in HEK293T cells. **(C)** Determination of the expression level of REV required for a reliable BRET measurement. HEK293T cells were transfected with increasing amounts of pRK5-Rluc8-EKAR_Nucl_-Venus and pRK5-Rluc8-EKAR_cyto_-Venus plasmids. BRET in cell population was expressed as a function of REV-fluorescence reporting the expression level of REV. Note that the BRET remained constant for a fluorescence of REV comprised between 200 and 1,300 photon counts which corresponds to cells transfected with 10–4,000 ng of plasmid per 100 mm diameter culture dish. **(D)** Determination of the expression level of REV required for efficient report of ERK activation. HEK293T cells transfected with increasing amounts of pRK5-Rluc8-EKAR_Nucl_-Venus and pRK5-Rluc8-EKAR_cyto_-Venus plasmids were stimulated with EGF and recorded over time. Note that EGF-induced ERK activity could be reported only in cells transfected with 10–100 ng of plasmid per 3,000,000 cells. **(E)** Basal BRET measured in HEK293T cells transfected with REV or REV_T/A_ inactive mutants. Each bar of the histogram represents the mean ± SEM of five independent experiments performed in triplicate. Note that the unphosphorylated form of REV, REV_T/A_, displays a lower BRET signal than the wild-type REV, highlighting a basal activity of ERK in our experimental conditions. The non-null net BRET of REV_T/A_ also emphasizes a basal BRET in the unphosphorylated (open) conformation of REV. **(C–E)** BRET in cell population were measured in the Mithras luminescence-fluorescence plate reader. Net BRET values were calculated by subtraction of the BRET obtained in cells transfected with Rluc8 alone, and multiplied by 1,000 to be expressed as milli BRET unit (mBu).

**Figure A1:**
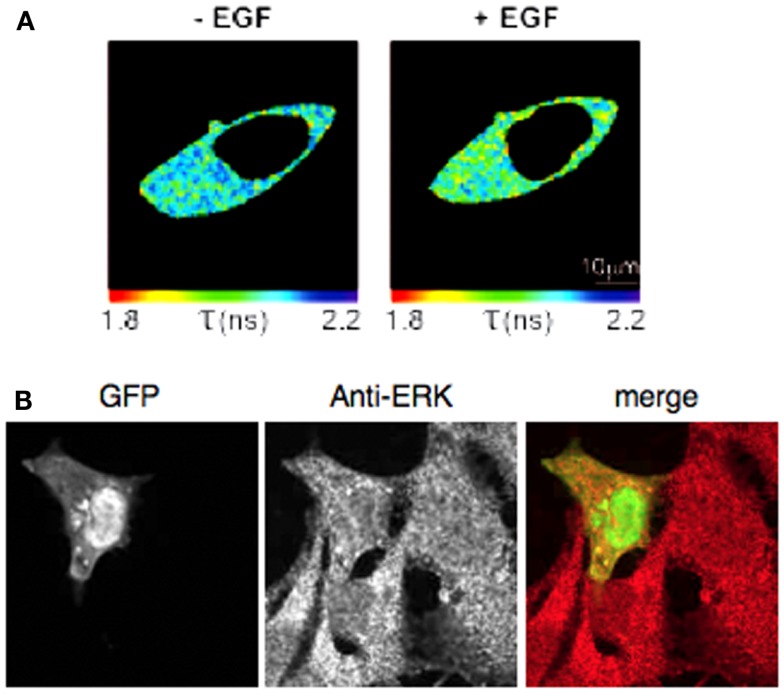
**(A)** Fluorescence lifetime images of a HEK293 cell expressing cytosolic EKAR before and after (8 min) addition of EGF. **(B)** REV-fluorescence (GFP) and ERK immunostaining (Anti-ERK) in HEK293 cells transfected with plasmids coding for cytosolic and nuclear REV constructs.

Extracellular signal-regulated Kinase Activity Reporter selectively and reversibly reported ERK activation in HEK293T cells after EGF stimulation (18). Using FLIM, we corroborated these data (Figure A1 in Appendix). However in our hands, this FRET-based sensor failed to report weaker biological stimuli also thought to modulate ERK activity. In order to detect subtle modulations of ERK activity with this biosensor, we hypothesized that the sensitivity of the assay would be improved by replacing the FRET donor and acceptor pair by BRET compatible entities. We therefore engineered a construct in which the *Renilla Luciferase* (Rluc8) and acceptor Yellow Fluorescent protein Venus are boxing the ERK substrate (Figure 1A), so-called “REV.” Because of the nuclear localization of the WW domain, REV expression was restricted to the nucleus when expressed in HEK293T cell (REV_nucl_, Figure 1B). We engineered a second plasmid containing an additional DNA sequence coding for a C-terminal nuclear export sequence, which resulted in cytoplasmic expression (REV_cyto_, Figure 1B). We controlled that REV transfection in HEK cells did not impair endogenous ERK expression. Both of them were broadly expressed in the cell (Figure A1B in Appendix). To characterize REV as a new ERK-biosensor, HEK293T cells were transfected with both nuclear and cytosolic plasmids, except when specified.

### Defining the experimental conditions to depict reliable BRET signals to report ERK activity

Rluc8-ERKsubstrate-Venus being an intra-molecular BRET biosensor, the stoichiometry of donor and acceptor entities is constant: one Rluc8 for one Venus per molecule. In its non-phosphorylated form, the reporter adopts an “open” conformation. Upon phosphorylation, conformational bending of the biosensor increases the proximity between the donor and acceptor in a “closed” conformation and induces a BRET-increase to report its phosphorylation by ERK. In an attempt to properly define the experimental conditions allowing reliable BRET signals, we first assessed the optimal expression level of REV. Three main criteria have to be fulfilled. This optimal expression must indeed be sufficient to be within the linear range of detection of the luminescence and fluorescence signals of the donor and acceptor entities respectively. However, too high a level of REV expression could induce non-specific inter-molecular BRET due to random collisions between proteins, which would bias the analysis. Finally, a minimal expression of REV would maximize its phosphorylation by endogenous kinases activation, while an excess of REV expression would preclude the phosphorylation of all molecules by ERK and thus prevent adequate detection of ERK activity. We thus performed several transfections of HEK293T cells with increasing quantity of REV expression plasmids (see Materials and Methods). The BRET signal expressed as a function of the fluorescence (which is proportional to the REV expression level) was constant, except for weak expression of REV revealing the limit of detection of the luminescent and fluorescent signals (Figure 1C). No inter-molecular BRET interference was detected for this range of protein expression. To assess the optimal quantity of DNA coding for REV to efficiently report ERK activation, we applied EGF on cells transfected with 0.1–4,000 ng of plasmid DNA coding for REV (Figure 1D). EGF-induced ERK activation was detected only in transfection conditions ranging from 10 to 100 ng of REV plasmid per 100 mm diameter cell dish (3,000,000 cells). In cases of ERK substrate (REV) stronger expression, only a small amount of REV might be phosphorylated by ERK activation, which prevents the optimal detection of ERK activity. In the following experiments, to favor REV-phosphorylation by endogenous kinases, we thus selected the transfection condition giving rise to the weakest expression level of REV within the linear range of BRET (corresponding to 20 ng of DNA per 100 mm cell dish, Figure 1C).

**Figure 2:**
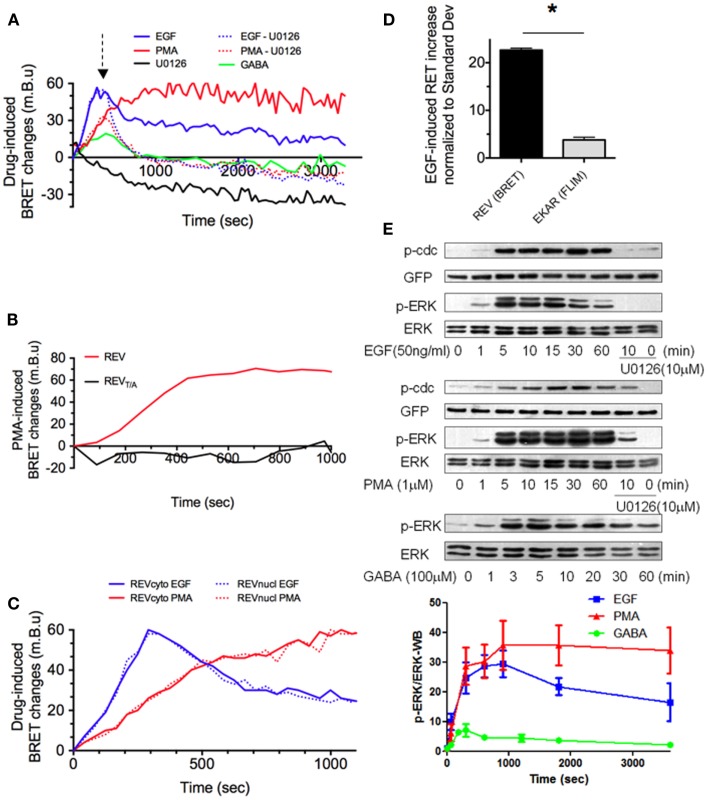
**REV, a sensitive BRET sensor of ERK activity**. **(A–D)** Drug-induced BRET changes of REV were measured in HEK293T cells population, in the Mithras luminescence-fluorescence plate reader. Cells were transfected with both cytosolic and nuclear constructs of REV **(A,B,D)** or only one of the two constructs **(C)**. In A, green line, GB1 and GB2 subunits of GABA-B receptor were co-transfected with REV. **(A)** EGF, PMA, and GABA application induced a BRET signal increase with different intensity and temporal profile, while U0126 decreased the BRET. The PMA or EGF-induced increase of BRET could be reversed by U0126 application (dotted lines, U0126 application is symbolized by the arrow, 300 s after PMA or EGF perfusion). **(B)** PMA-induced BRET changes was measured in HEK293T cells transfected with REV or REV_T/A_. The absence of effect of PMA in REV_T/A_-transfected cells validated the specificity of REV to report ERK activation. **(C)** BRET changes induced by PMA (red) or EGF (blue) were measured in the cytosol (full lines) or nucleus (dotted lines). **(D)** Sensitivities of EKAR (FRET/FLIM-based reporter) and REV (BRET-based reporter) were compared by normalizing the EGF-effect to the standard deviation in their respective technology. **(E)** ERK, phospho-ERK (p-ERK), REV (GFP), and phospho-REV (p-cdc) staining quantified by western blot in HEK293T cells transfected (GABA – green line) or not (EGF and PMA conditions, blue and red line) with GB1 and GB2 subunits of GABA-B receptor before and up to 60 min after drug application. When specified, following 10 min of EGF or PMA incubation, U0126 was added for 50 min. p-ERK/ERK ratio were calculated in each column and normalized to the p-ERK/ERK ratio measured before stimulation (*t* = 0). Each point of the graph below represents the mean ± SEM obtained from three individual experiments, for each time condition.

HEK293T cells expressing REV displayed a mean basal net BRET signal of 255.55 ± 1.15 milli BRET units (mBu, Figure 1E). This basal BRET signal might report either a sufficient proximity between the donor and acceptor entities in the non-phosphorylated conformation of REV and/or a non-null phosphorylation of REV due to a basal activity of ERK. To discriminate between these two possibilities, we mutated the MAPK phosphorylation site in the Cdc25C peptide (Thr-to-Ala substitution, REV_T/A_) (18). The basal BRET displayed by the REV_T/A_ mutant was significantly weaker (225.64 ± 2.6 mBu) than the wild-type biosensor (Figure 1E), highlighting a basal ERK activity. This was further confirmed by perfusion of the ERK pathway inhibitor U0126 (10 μM), which induced a BRET decrease of −32.79 ± 0.68 mBu in REV-transfected cells (Figure 2A). The remaining basal BRET with REV in presence of U0126 and the non-null BRET displayed by the REV_T/A_ mutant both evidenced a proximity between Rluc8 and Venus in the non-phosphorylated form of REV compatible with intra-molecular BRET.

**Figure 3:**
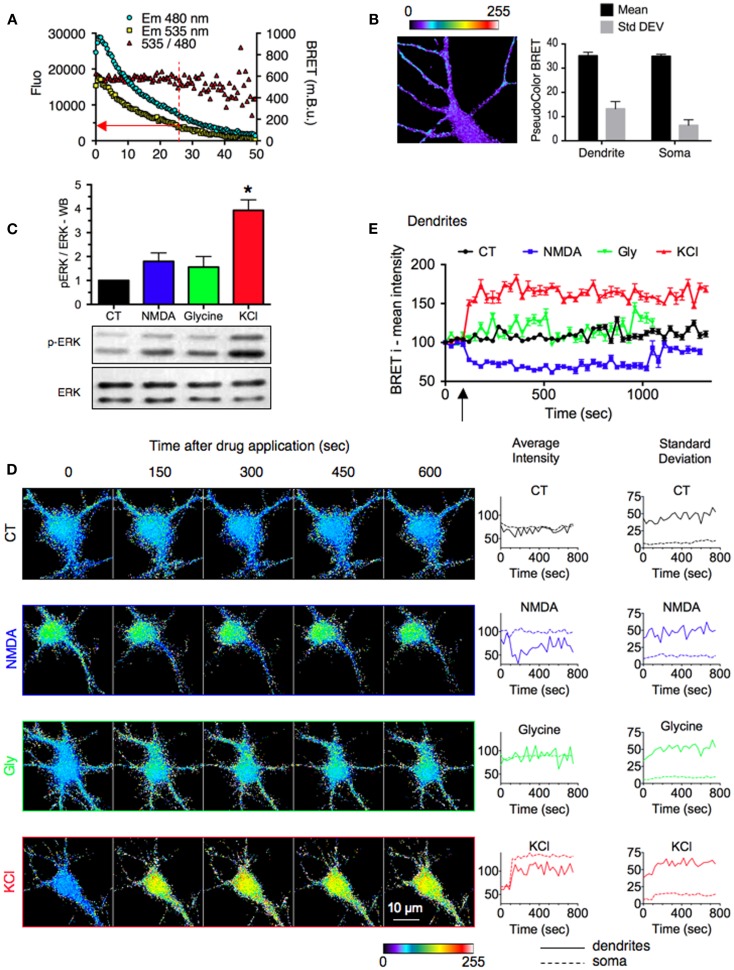
**Stimulus-specific responses of ERK signaling reported by REV in neuronal sub-cellular compartments**. **(A)** Evolution of Em480, Em535, and 535/480 ratio signals over time. Sequential BRET images on neurons expressing REV were acquired for 50 min. The red arrow indicates the cut-off value for luminescence intensity below which BRET fluctuations prevented adequate measurements. **(B)** Basal signal resulting from the overflow of the energy donor output into the energy acceptor detection channel. Neurons were transfected with RE (REV construct without Venus). Left, representative BRET image. Right, BRET intensity (mean) and distribution (Standard Deviation) in soma and dendrites. Each bar of the histogram represents the mean ± SEM obtained from three to six neurons and seven regions per neuron. **(C)** ERK and phospho-ERK (p-ERK) staining quantified by western blot in hippocampal neurons stimulated or not (black) with NMDA (50 μM, blue), Glycine and strychnine (200 and 1 μM, respectively, green), or KCl (50 mM, red) for 10 min. **(D)** Real-time BRET imaging of four representative hippocampal neurons transfected with REV, in the stimulated conditions described in **(A)**. Changes of average intensity and standard deviation over time were measured on the soma and dendritic shaft of each image. **(E)** Intensities of BRET signals recorded on dendrites of neurons in BRET images over time. Each point of the graph represents the mean ± SEM obtained from three to six neurons and seven regions per neuron, for each time condition.

### REV efficiently and specifically reports ERK activity in living cells

To assess the efficiency of REV to report ERK activation, REV-expressing cells were stimulated with EGF (50 ng/ml) or phorbol myristate acetate (PMA, 1 μM) to strongly activate ERK signaling. EGF application induced a transient BRET-increase that was maximal 5 min after stimulation (Figure 2A). Conversely, the PMA-induced BRET-increase was slower (maximal 15 min after stimulation), but stable for more than 1 h (Figure 2A). Both EGF- and PMA-induced BRET-increases could be reversed by application of the ERK inhibitor U0126 (10 μM, dotted lines, Figure 2A). Furthermore, no BRET changes were recorded upon PMA application in HEK293T cells transfected with the mutant REV_T/A_, confirming the specificity of the biosensor to report ERK phosphorylation specifically (Figure 2B). Under EGF- or PMA-induced ERK stimulations, no difference was found between nuclear and cytosolic REV reporters when expressed separately (Figure 2C). These drug-specific profiles of ERK activation reported by REV were in accordance with western blot (WB) analyses to reveal the phosphorylated form of endogenous ERK and transfected REV (using Anti-ERK and Anti-phospho-Thr-48-cdc25c antibodies, respectively) (Figure 2E). However, we noticed a slight shift in time of the maximal ERK activity using WB compared to real-time BRET experiments. This temporal shift might be the result of the WB experiment’s lack of precision due to the use of detergent to lyse cells and solubilize proteins. The BRET sensor thus appeared to be an accurate reporter of ERK activity, with the main advantage of reporting the modulation of ERK activity in real time in the same living cells.

To characterize the sensitivity of this BRET-based sensor, we measured the ratio of EGF-induced increase in REV BRET (57.32 ± 1.03 mBu, mean ± SEM obtained from three individual experiments) over the standard deviation of BRET signals in basal condition (2.53 ± 0.14 mBu, mean of Standard Deviation ± SEM obtained from three individual experiments) and found that the EGF-induced REV BRET-increase was 22.6 times higher than the standard deviation of the BRET. Similar quantification was performed with EKAR, the most sensitive FRET-based sensor so far published. FLIM experiments showed an EGF-dependent decrease in EGFP fluorescence lifetime of 0.0560 ± 0.0082 ns for a 0.0147 ± 0.0017 ns standard deviation of the signal in basal condition. The EGF-induced FRET increase was therefore 3.8 times higher than the standard deviation. To place emphasis on the higher sensitivity of REV, we normalized EGF-induced EKAR and REV signals modulation to the standard deviation of FRET and BRET signals respectively (Figure 2D).

The high sensitivity of REV suggested the possibility of reporting subtle modulations of ERK activity with this BRET-based sensor, which we failed to do with EKAR by FRET/FLIM. For example, GABA-B receptor stimulation in HEK293T cells transfected with GB1 and GB2 subunits induced a shorter and weaker increase in ERK activity as shown by WB (Figure 2E). This transient weak activation of ERK activity could successfully be reported in real time with REV in living cells (Figure 2A). Taken together, these results identify REV as a sensitive biosensor to report ERK activity in living cells, providing that its expression level was sufficient to reliably read the BRET signals but weak enough to allow its phosphorylation by endogenous ERK.

### REV reports stimulus-specific responses of ERK signaling in neuronal sub-cellular compartments

In light of the good sensitivity of REV, we performed BRET imaging experiments in hippocampal neurons to depict the spatio-temporal dynamics of ERK activation. First, as mentioned previously for BRET in cell population, a sufficient level of expression is needed in order to be in the linear range of detection of luminescence and fluorescence. This consideration also applies to BRET imaging to obtain an accurate evaluation of BRET signals. To determine a cut-off for the minimal level of luminescence emission required for an accurate BRET ratio measurement, we measured the evolution of Em480, Em535, and BRET intensity over time (Figure 3A). The BRET signal over time was constant until the luminescence decreased fewer than 4,000 counts where the fluctuation between successive BRET readings strongly increased. Accordingly, in order to exclude these non-reliable pixels of weak emission, we applied an exclusive threshold on the Em480 image before carrying out the pixel-by-pixel division of Em535/Em480 to obtain the BRET image.

One of the major difficulties in establishing BRET imaging is to distinguish the signal originating from the transfer of energy from that resulting from an overflow of the energy donor output into the energy acceptor detection channel. To control for this basal signal, we engineered a REV construct without Venus (RE). Neither the mean BRET signal nor the standard deviation, were found to be significantly different in the soma and dendritic areas (Figure 3B). As expected, RE displayed a homogenous and weak basal BRET, independent on the luminescence level. This validated the accuracy of our experimental conditions.

In neurons, ERK activity is modulated by the neuronal activity and has been involved in opposing cellular processes such as long term potentiation (LTP) or depression (LTD) of the synaptic transmission (26). Several studies have shown that ERK activity is indeed regulated by different stimuli, among which we chose three examples. (1) KCl depolarization induces sustained ERK activation in hippocampal neurons (27, 28). (2) Selective extrasynaptic NMDA receptor activation (as well as NMDA bath application) does not activate ERK pathways, whereas (3) synaptic NMDAR activation – to induce a chemical LTP – induces ERK activation (29). WB experiments indeed confirmed these findings but only KCl application was found to be significantly different from the non-stimulated condition (Figure 3C).

To further decipher stimulus-specific responses of ERK signaling we used REV to report in living neurons the location, duration, and strength of ERK activity in sub-cellular compartments. Compared to control condition (buffer perfusion), KCl application (50 mM, 10 min) induced a sustained increase in BRET intensity both in soma and dendrites, reporting ERK activation upon depolarization (Figures 3D,E). The standard deviation increased in dendrites, suggesting an important clusterization of ERK activity induced by KCl application (Figure 3D). Conversely, NMDA bath application (50 μM, 10 min) decreased the BRET signal intensity and this inhibition of ERK activity could be seen in dendrites only (Figure 3D). Thus, while a global approach such as WB experiment failed to report significant modulation of ERK activity by NMDA (Figure 3C), the possible sub-cellular analysis with the BRET-based sensor highlighted a dendritic significant inhibition of ERK activity (Figure 3E). In presence of glycine and strychnine (200 and 1 μM, respectively, for 3 min) the BRET intensity slightly increased in soma and dendrites as well as standard deviation in dendrites, compared to control condition (Figure 3D). However, in contrast to the KCl-induced increase and NMDA-induced decrease, this Glycine-induced modulation of BRET signals was not significantly different from the control condition when several experiments were pooled (Figure 3E).

## Discussion

In the present work we have engineered and tested the first BRET reporter of ERK activity, REV. REV selectively and reversibly reported ERK activity after EGF or PMA stimulation in HEK293T cells and following changes in neuronal activity in hippocampal neurons. REV therefore allows the analysis of ERK signaling in time and space in living cells. We here defined the experimental conditions required to use this BRET-based sensor and achieved a proof of principal study to highlight several advantages of REV to improve the detection of ERK activity. Finally REV was used to point out spatio-temporal profiles of ERK activity induced by different stimuli, so far unrevealed by other technologies.

Our results highlight the need to carefully control the expression level of the biosensor to reach optimal conditions and report subtle modulations of endogenous kinases activity. The expression level of the reporter has to be sufficient to allow a reliable detection of the light emitted by the BRET donor and acceptor. However the reporter expression level must not be excessive to efficiently report the activity of endogenous kinases. We precisely defined the limits of REV expression to report ERK activation by EGF application in HEK cells. Similarly, in neurons, we determined a cut-off for the minimal level of luminescence emission required for an accurate BRET ratio measurement. In order to exclude these non-reliable pixels displaying low luminescence value, we applied an exclusive threshold on the Em480 image before dividing Em535/Em480 pixel-by-pixel to obtain the BRET image. The mean BRET values as well as the distribution (Standard Deviation) of BRET obtained from a sensor expressing only the BRET donor entity was found to be homogenous both in dendrites and soma. This result confirmed the accuracy of our experimental conditions. Once again, in order to optimize the number of REV molecules that would be phosphorylated to report endogenous ERK activation, we chose neurons with the weakest fluorescence, i.e., those in which the expression level of REV was low. We further corroborate our BRET experiments by WB experiments to reveal the phosphorylation of the reporter as well as ERK auto-phosphorylation induced by ERK stimulation in different conditions. The consistence of the results obtained with BRET and WB, further validated our experimental conditions. Nevertheless, it is important to mention that the expression level of REV defined herein may not satisfy every kind of ERK activation. In low ERK activation conditions, only a small amount of REV would be phosphorylated by ERK activation, which would prevent the optimal detection of ERK activity. This might be the reason why REV failed to reliably report one of the weakest modulations of ERK activity we found by WB, the glycine-induced modulation.

Rluc8-ERKsubstrate-Venus selectively reported ERK activity. This was assessed by point mutation of the REV-phosphorylation site, and by the use of ERK inhibitor, U0126. U0126 indeed put emphasis on a basal ERK activity and was also effective in reversing EGF- and PMA-induced BRET-increase. Intriguingly, this reversion did not reach the BRET levels obtained with U1026 alone. WB experiments confirmed that a pre-stimulation of ERK by PMA led to ERK auto-phosphorylation and REV-phosphorylation which were not totally abolished by a subsequent 50 min incubation of U0126, Figure 2E. Thus U0126 has a higher potency to decrease ERK substrate phosphorylation when applied alone. The residual phosphorylation of ERK or REV following sequential application of PMA and U0126 might come from the fact that once phosphorylated the REV sensor may be a poor substrate for phosphatases and only a part of phosphorylated REV would return to an inactivated state following U0126 incubation. Moreover the dephosphorylation rate of REV may be different in cells incubated only in U0126 compared to cells with high activation of ERK (EGF/PMA) since phosphatases activation could be affected differently. Nor can one exclude a non-specific action of U0126 and some phosphorylation of the ERK sensor, due not only to ERK but to other kinases not inhibited by U0126. However, no BRET changes were recorded upon PMA application in HEK293T cells transfected with the mutant REV_T/A_, arguing in favor of the specificity of the biosensor to report ERK phosphorylation.

This proof of principal study highlights several advantages of REV to improve the detection of ERK activity. One obvious benefit coming from the BRET-based sensor is the possibility to work on living cells and report the kinetics of ERK activation in real time on the same cell. FRET-reporters present the same advantage, but this BRET biosensor displayed a higher sensitivity. Consequently, compared to classical WB experiments, REV avoids the variability of ERK activity measurements from different pools of cells over time. Moreover, BRET imaging reported a variability of ERK activation status in a cell population. This cellular precision indeed extend the need for biosensors such as REV, that could be used in real time on the same living cell rather than pools of cells in which the mean signal would not change over time. For example, the basal ERK activation status could indeed have been a limiting factor to see the NMDA effect (with a lower basal ERK activity, we may have missed the NMDA-induced decrease of BRET). This comment also applies to the sub-cellular location of the activation of a signaling pathway. With BRET imaging we were able to highlight subtleties in sub-cellular activation of ERK that we missed with a more general approach such as WB experiments (see again the NMDA-induced modulation of ERK reported by WB compared to BRET, Figure 3). Even within sub-cellular compartments, BRET imaging enables the characterization of the distribution of BRET signals by measuring the standard deviation between pixels of the same area. For example, the high standard deviation seen in dendrites of neurons expressing REV, even in basal condition, indicates a spatial clusterization of the ERK activity along dendrites. The cellular and sub-cellular precision thus increases the sensitivity of the assay compared to global methods. Finally, one fundamental property of REV is its high reproducibility. We averaged the mean BRET intensity as well as the BRET distribution (Standard Deviation) on several areas drawn in identical compartment and stimulating conditions. Both averages displayed small SEM, highlighting the reproducibility between similar areas, and the consistence and accuracy of the results obtained with the BRET sensor.

In this first study using REV we pointed out spatio-temporal profiles of ERK activity induced by different stimuli, some of them so far unrevealed by other technologies. Firstly, REV displayed a basal BRET signal, which could be decreased by mutation of the REV-phosphorylation site. This highlighted a basal ERK activity. In neurons, this basal ERK activation was spatially clustered along dendrites. One direct consequence of REV reporting basal activity of ERK is to allow the detection of decreases in ERK activity. Secondly, in HEK cells, we found similar kinetics for cytoplasmic and nuclear ERK activation, which confirms the data obtained with FRET sensors by Harvey et al. (18). The exact molecular mechanisms underlying ERK translocation to the nucleus are still unknown and are the subject of interesting debate. This further highlights the need for real-time sensors for ERK activity. Our results suggest that regulation of ERK activity is similar in the somatic cytoplasm and nucleus, possibly because of a rapid diffusional exchange between the two compartments (30). Thirdly, in neurons, we demonstrated that the strength, the location, and the moment of ERK activation depends on the nature of the stimuli, highlighting that ERK is not simply switched on and off, but rather that acute modulation of ERK will encode the nature of the initial stimulus and specify the consequence of its activation. REV is therefore well suited to address such an important biological issue, not only in neurons but also in any other cell type.

Several experimental adaptations may improve the sensitivity of this BRET-based biosensor. First we will increase the length of the central flexible linker to increase the distance between the donor and acceptor entities in the basal (non-phosphorylated) condition. This should decrease the basal BRET signal still detected with REV in presence of U0126 (ERK inhibitor), or with the mutant of REV_T/A_ that cannot be phosphorylated (REV_T/A_). Accordingly, by decreasing basal BRET we expect a larger window for BRET modulations induced by ERK activation, which could help in reporting subtle variations. Second, the use of BRET compatible entities for the third generation of BRET [BRET3, with Rluc8 as donor and mOrange as acceptor (31)] should also increase the sensitivity of this assay. Indeed, the improved spectral resolution between Rluc8 and mOrange compared to Rluc8 and Venus will minimize the bleed-through of the donor fluorescence into the acceptor detection channel, decreasing the background signal and therefore increasing the sensitivity of BRET measurements. Accordingly, we replaced Venus by mOrange in the BRET-based sensor to obtain REO (Rlu8-ERKsubstrat-mOrange). Ongoing experiments are testing this reporter, which will further present the advantage to be compatible with BRET1-based reporters of other signaling pathways. Another way to improve this sensor could be provided with a higher luminescence signal by pixel without increasing the concentration of biosensor per cell, using a new smaller and brighter luciferase called Nanoluc (Promega). Finally, because the level of expression of the BRET-based sensor is a limiting factor to accurately report ERK activity, we wish to control the expression of the biosensor by endogenous promoter in order to be in harmony with the expression levels of ERK targets.

To conclude, this efficient ERK sensor has to be added to the library of BRET-based sensors so far generated (32) and will help understanding integrated signaling dynamics in time and space (33).

## Conflict of Interest Statement

The authors declare that the research was conducted in the absence of any commercial or financial relationships that could be construed as a potential conflict of interest.
